# A new epoxy for plastination: feasibility and applicability analysis of the conservation of biological tissues

**DOI:** 10.1590/1414-431X2025e14839

**Published:** 2025-10-17

**Authors:** Y.F. Monteiro, E.A. Patrício, L.E.S.B. Campos, K. Soares, B.V. Nogueira, A.S. Bittencourt

**Affiliations:** 1 Laboratório de Plastinação, Departamento de Morfologia, Centro de Ciências da Saúde, Universidade Federal do Espírito Santo, Vitória, ES, Brasil; 2 Programa de Pós-Graduação em Biotecnologia, Centro de Ciências da Saúde, Universidade Federal do Espírito Santo, Vitória, ES, Brasil; 3 Instituto Federal do Espírito Santo - Campus Vitória, Vitória, ES, Brasil

**Keywords:** Shrinkage, Plastination, Silicone, Preservation, Viscosity

## Abstract

Plastination is a technique for preserving biological tissues, in which body fluids are replaced by a curable polymer. Epoxy resin is used for 2-5 mm sections of anatomical segments, with the German-made Biodur^®^ E12 being the best known and most widely used resin. A few alternative epoxies can be used in the technique, but research should be developed to identify options that are cheaper and less bureaucratic to acquire. This study aimed to find, adapt, and apply an alternative epoxy resin formulation and its curing system for the plastination process as a potential substitute for Biodur^®^ E12. The methodology was divided into the search of a resin for national commercialization in Brazil, the development of the final formulation, the testing of its use in plastination, and the evaluation of the resin and final specimens. From market research, E48 epoxy (brand not disclosed) was selected, and its formulation was changed with the addition of a plasticizer for use in impregnation. A total of 150 Wistar rat cross-sections were plastinated with the control polymer (E12) and with the developed test resin (E48). Based on the positive results of the shrinkage analysis (no statistical difference) and confocal and stereoscopic microscopy, it was concluded that the modified E48 is a great alternative to E12.

## Introduction

Plastination, a technique for the preservation of biological tissues, was developed in 1977 by the physician Gunther von Hagens, and its basic principle is the replacement of body fluids with a reactive polymer. The technique is divided into the steps of fixation, dehydration, forced impregnation, and curing. After plastination, the biological specimens are inert, non-toxic, dry, resistant, and durable and can be used in the most diverse areas, such as teaching, research, and social programs (1).

The most used polymer categories in the process are silicone, polyester, and epoxy (2), which determine the mechanical and optical properties of the final specimen (1). Epoxy resin is mainly used for sections of 2-5 mm thickness of any part of the body or biological tissue, producing a more rigid, crystalline, and semi-transparent final specimen (3).

The main component of the epoxy resins used in plastination is diglycidyl ether of bisphenol A (DGEBA), which results from the reaction between bisphenol A and epichlorohydrin. DGEBA epoxy resins become thermoset materials from the curing reaction with various classes of chemical compounds, of which aminic compounds are the most common ones. Epoxy resins have minimal contraction during the curing or polymerization process and have good resistance to heat and chemical attack (4).

The German company Biodur^®^, founded by the inventor of plastination, markets the most widely used and well-known polymers for the technique, which are specially developed and tested for this purpose (5). Their epoxy resins differ in terms of applications, and optical and mechanical characteristics. Currently, there are two options available: E12 and E13, with E13 having a greater resistance to yellowing of the resin after curing. However, E12 is the most commonly used because it is a product that has been on the market for a long time and is easy to use.

Other large companies from different countries also market products used in plastination, which facilitates acquisition by plastinators around the world and stimulates research and development of polymers with different optical and mechanical characteristics for specific purposes in plastination (6). In Latin America, for example, customs and freight costs can double or triple the value of products imported from Germany.

Therefore, investigating the use of an alternative epoxy resin to the reference resin (Biodur^®^ E12) for the plastination of biological tissue slices (2-3 mm) may offer a cheaper and less bureaucratic option for epoxy-based plastination in countries of Latin America and other continents. In addition, the results can be a guide for the search for alternative epoxies in other countries.

This study aimed to explore, adapt, and apply an alternative epoxy resin formulation and its curing system for plastination, evaluating its potential as a substitute for Biodur^®^ E12.

## Material and Methods

### Alternative epoxy screening and initial examination

Initially, we screened for an epoxy resin and curing agent with physicochemical and reactional characteristics for application in the production of thin plastinated sections (2-5 mm) in the Brazilian market, using the Biodur^®^ E12 resin as a reference. For the initial evaluation, resin characteristics were obtained from technical sheets, and the main prerequisites were observed: bisphenol A and epichlorohydrin-based resin, high transparency, and viscosity lower than 4,000 mPa.s. Resin E48 and hardener E5 were selected. The trade name and manufacturer of E48 and E5 are withheld due to patent and commercial registration reasons.

Once the potential alternative resin was found, a phthalate-based plasticizer was tested to extend the gel and curing time of the resin-hardener mixture, since the curing time indicated by the technical sheet was two to three hours. This test was essential to establish a reactive mixture that provides a working time of over 10 h, as after the addition of the hardener to the resin, the mixture quickly begins polymerizing to gel and curing states (7,8). According to previous research and our preliminary tests, decreasing the concentration of the hardener to extend the curing time affects the complete curing of the epoxy resin, and, therefore, the stoichiometry of the polymerization reaction must be respected (9,10). The plasticizer compound is not disclosed for commercial reasons.

The gel/curing time test *vs* plasticizer concentration was serialized using six concentrations of plasticizer in the reaction mixture with the resin: 0, 5, 15, 25, 30, and 35% m/m, respecting the hardener concentration suggested by the manufacturer (43% relative to the resin). For this, serial masses of 40 g of E48 resin and plasticizer were separated in the chosen proportion, and the corresponding amount of E5 hardener was added under homogenization with a glass rod for one minute. The time elapsed from mixture to gel state and complete curing was recorded. For the control group, the gel and curing time for the E12 resin was measured in the proportion recommended by the manufacturer Biodur^®^ (100:28 m/m). All gel/curing time tests were performed in duplicate for each sample and the mean time was calculated.

### Plastination of samples with the alternative epoxy

This research was approved by the Animal Ethics Committee of the Federal University of Espírito Santo (CEUA-UFES), under No. 31/2019.

Ten freshly euthanized Wistar rats were enclosed in polyurethane and frozen in a freezer for five days to ensure even freezing. After that, 2-mm-thick cross-sections were sequentially obtained with a band saw (Skymsen SSI No. 1974, Brazil). A total of 150 high-quality, usable slices were obtained. The sections were then organized between perforated plastic grids and cotton fabrics to facilitate handling.

Sections were dehydrated with three successive weekly immersion baths in cold pure acetone in freezers (−25°C). The acetone concentration remained above 99.5% at the end of the last bath, which is the requirement for the stage to be considered finished.

After dehydration, the 150 slices were divided into experimental groups E12 and E48 (75 slices each), and forced impregnation was performed in three replications, i.e., 25 slices from each group. The sections were immersed in the newly prepared reactive mixtures, with the E12 mixture consisting of E12 resin + E1 hardener in the proportion 100:28 m/m in the control group and the E48 epoxy mixture that varied according to the results obtained in the previous impregnation, as shown in Table 1. In the second and third impregnation tests, the same impregnation and curing mixture was used to verify the repeatability of the results.

**Table 1:** Experimental groups (E12 and E48), number of samples, and proportions of reactive impregnation mixtures according to the impregnation repetition.

Replication	Resin	N sample	Impregnation mixture (m/m)	Curing mixture (m/m)
1st test	E12	25	E12 + E1 (100:28)	E12 + E1 (100:28)
	E48	25	E48 + plasticizer + E5 (65:35:28)	E48 + plasticizer + E5 (65:35:28)
2nd test	E12	25	E12 + E1 (100:28)	E12 + E1 (100:28)
	E48	25	E48 + plasticizer + E5 (65:35:28)	E48 + E5 (100:43)
3rd test	E12	25	E12 + E1 (100:28)	E12 + E1 (100:28)
	E48	25	E48 + plasticizer + E5 (65:35:28)	E48 + E5 (100:43)

In each forced impregnation test, the control epoxy (E12) and the alternative epoxy samples were placed together inside a medium circular vacuum chamber (Biodur^®^, 100 L capacity), and a slowly increasing vacuum was applied. The vacuum adjustment was performed in a way that the impregnation lasted 8 h (8), with an intense and constant pattern of acetone bubbles. The impregnation of the experimental groups started at a pressure of 200 mmHg, and, in the first hour of impregnation, the pressure was reduced to 100 mmHg. Over the following six hours and 40 min, the pressure was reduced by approximately 5 mmHg every 20 min. As a result, at approximately one hour and 20 min before the end of impregnation, the chamber’s air inlet valves were completely closed, reaching the maximum vacuum of the pump (2 mmHg). The stage was completed when the total impregnation time had elapsed and a marked reduction in bubble formation was observed.

The curing step was performed using the standard sandwich method. The sections were placed with the curing mixture of each epoxy (Table 1) between acetate sheets and a glass slide. After that, the assembly remained at room temperature for 72 h and was then transferred to an oven at 45°C for another 48 h before being dismantled.

### Evaluation of sections plastinated with different epoxies

The sections produced with the E48 epoxy test and the E12 control group were compared considering compatibility with the technical steps and quality of the final product. The main parameters were the behavior of the test resin in the different steps: forced impregnation, chemical curing, and sandwich dismantling compared to the control group.

For the evaluation of the final products, the general aspects of the thin epoxy sheets were observed, such as the complete curing of the polymers, malleability, the intended crystallinity of the different types of tissues present in the slices, and the yellowing of the epoxy plates.

### Evaluation of tissue shrinkage

**Figure 1:**
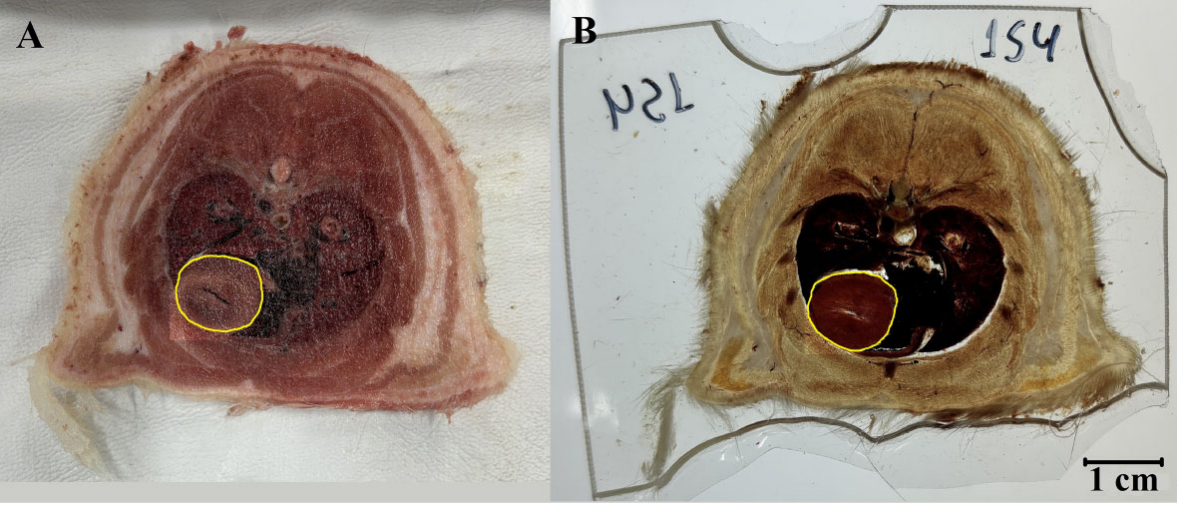
Measurement of the area (cm^2^) of the heart in a section of the thorax before forced impregnation (**A**) and after curing (**B**) by the ImageJ program. The yellow demarcation and the measuring scale (one centimeter) are shown in the software for calculation.

The shrinkage of the biological tissues was calculated by the difference between the initial area (before impregnation) and the final area (after curing). Nervous, renal, hepatic, splenic, cardiac, and pulmonary tissues were evaluated. The areas were measured with the free software ImageJ (NIH, USA) on only one side of the section from standardized photographs and under the same conditions of distance, angulation, and lighting. The software estimates the area from the pixel count of the photos, having as parameter an informed scale (Figure 1). The measurement methodology was described by Monteiro et al. (11).

From the measurements, the shrinkage percentage per area of analyzed tissue was estimated using the formula: Shrinkage (%) = (area before impregnation (cm^2^) – area after curing (cm^2^)) × 100 / area before impregnation (cm^2^).

In the case of lobulated/segmented organs, segments with well-defined limits in the before and after photographs were considered for measurement.

Slices were excluded from the tissue shrinkage analysis based on the following criteria: absence of target tissues, considerable tissue damage, and color changes (darkening) in the regions of interest that hindered clear observation of tissue boundaries.

For the statistical analysis, the Shapiro-Wilk and Levene tests were used to test for normality and homogeneity of the variances, respectively. The mean shrinkage percentage data of the experimental groups were analyzed with the paired Student's *t*-test and one-way analysis of variance (ANOVA). A 5% significance level was adopted.

### Stereoscopic and confocal fluorescence microscopy

To verify the possible application of E48 epoxy in microscopic research - one of the main uses of plastination with epoxy (E12) - a general qualitative evaluation of the tissues was carried out. For comparative analysis between experimental groups E12 and E48, the same tissue sections used for shrinkage analysis were examined with a Leica E48 HD (Germany) stereomicroscope with 8× magnification, and the images were recorded electronically. Subsequently, the Zeiss LSM 880 Airyscan (Germany) confocal microscope was used, with the laser set at a wavelength of 488 nm and under a 10× magnification objective, and the images were also electronically recorded.

## Results and Discussion

### Alternative epoxy screening and initial verification

Table 2 shows the initial characteristics of the E48 resin and the E5 hardener and the E12 epoxy and E1 hardener characteristics obtained from the technical sheet provided by Biodur^®^ and the manufacturer of the alternative resin.

**Table 2:** Basic characteristics of Biodur^®^ resin and hardener (E48 and E5) and testing material (E12 and E1).

Characteristics	E48 epoxy	E5 hardener	E12 epoxy	E1 hardener
Main component	Bisphenol A - epichlorohydrin	Modified cycloaliphatic amine	Bisphenol A - epichlorohydrin	DMDC
Appearance	Viscous colorless liquid	Colorless liquid	Viscous colorless liquid	Colorless liquid
Viscosity (25°C)	500-1,600 mPa.s	60-80 mPa.s	3,000-4,000 mPa.s	140-150 mPa.s
Resin/hardener ratio	100:43		100:28	
Gel time	≅1 h		≅16 h	

DMDC: 2,2'-Dimethyl-4,4'-methylenebis (cyclohexylamine). The information was obtained from the technical data sheets provided by the manufacturers.

The alternative products had similar basic characteristics to the reference, such as visual appearance and general formulation. Although the viscosities differ between manufacturers, this factor is probably not of great relevance in plastination, since impregnation occurs in thin slices (2-3 mm) with a large contact surface.

The resin/hardener ratio and the gel time differed between the materials. The E12/E1 formulation was developed for impregnation in the plastination protocol, while the tested product was developed for other applications and purposes, showing a faster curing time (approximately three hours). Therefore, a plasticizer/diluent was used to prolong the gelation and curing of the resin, allowing an efficient impregnation and, consequently, a better result of the plastinated product.

Table 3 shows the results of gel/curing time test *vs* concentration of the plasticizer used to define the reactive impregnation mixture. The percentage of hardener that was added was calculated from the new resin (epoxy and plasticizer). The amount of hardener added to the E48 samples was calculated maintaining the 43% proportion suggested by the manufacturer for the pure resin/hardener ratio, without accounting for the plasticizer, and, therefore, the percentage of added hardener in Table 3 was calculated from the new formulation, that is, from the mixture of the resin with the addition of the diluent.

**Table 3:** Gel and curing times of E48 resin and E5 hardener with different proportions of the plasticizer (samples one to six) and of the E12 and E1 mixture as recommended by Biodur^®^ (sample seven).

Sample	Epoxy (%)	Plasticizer (%)	Hardener (%)	Gel time	Curing time
1	100	-	43	1 h	3 h
2	95	5	40.8	1 h 35 min	14 h
3	85	15	36.6	2 h 50 min	24 h
4	75	25	32	5 h 45 min	36 h
5	70	30	30	8 h 30 min	48 h
6	65	35	28	11 h	60 h
7	100	-	28	12 h	24 h

Ideally, the process of impregnation and assembly of biological samples for curing should be completed in approximately 10-12 h, and, therefore, the gel point of the resin should occur within this time to enable the completion of the steps. The complete epoxy curing and, consequently, the complete plastination of specimens, should not exceed 72-96 h.

All tested samples with diluent variation were cured (dry to touch), although their stiffness varied. By applying pressure to the touch, it was noted that the higher the concentration of diluent, the lower the stiffness of the cured resin.

Sample six demonstrated promising characteristics for use in plastination, considering its impregnation time before entering the gel state and its complete curing in 60 h (Table 3). Therefore, this formulation was selected for testing in the impregnation of biological samples.

### Evaluation of plastinated sections

A total of 150 slices of Wistar rats, divided into three moments of impregnation, were plastinated for the two experimental groups (E12 and E48). In all impregnation replications, 25 sections of each resin were processed in the same vacuum chamber and under the same conditions of temperature and impregnation speed.

**Figure 2:**
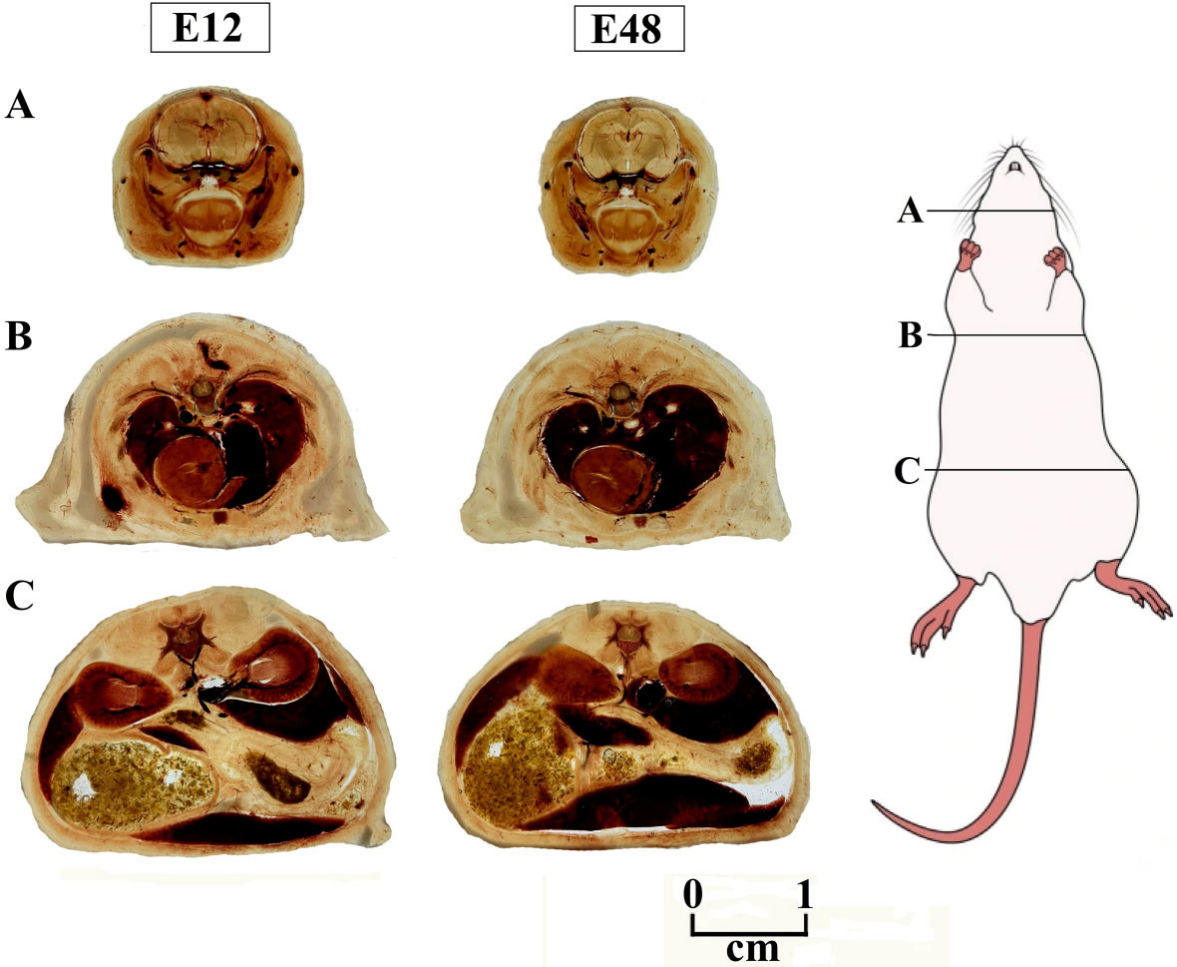
Head (**A**), thorax (**B**), and abdomen (**C**) slices of Wistar rats plastinated with E12 and E48 epoxy resins with plasticizer (ratio 65:35).

The slices plastinated with E12 and E48 (from all three impregnation tests) were very similar in contrast and crystallinity. In addition, all of them were dry to the touch, indicating the complete curing of the resins. It was not possible to visually distinguish between the experimental groups (Figure 2).

For both epoxies, the most vascularized tissues, such as the lung, liver, and heart, had a darker color and lower crystallinity compared to other tissues.

As expected, the sections plastinated with the control epoxy (E12) were easily assembled and disassembled from the curing sandwich, without sticking to the acetate. Yellowing was also clearly observed from the 6th month of completion, even though the pieces were stored in a dark box and handled sporadically under mild lighting and temperature conditions (following laboratory routine).

In plastination tests one to three with the E48 resin, no visual differences were observed between the biological tissues.

**Figure 3:**
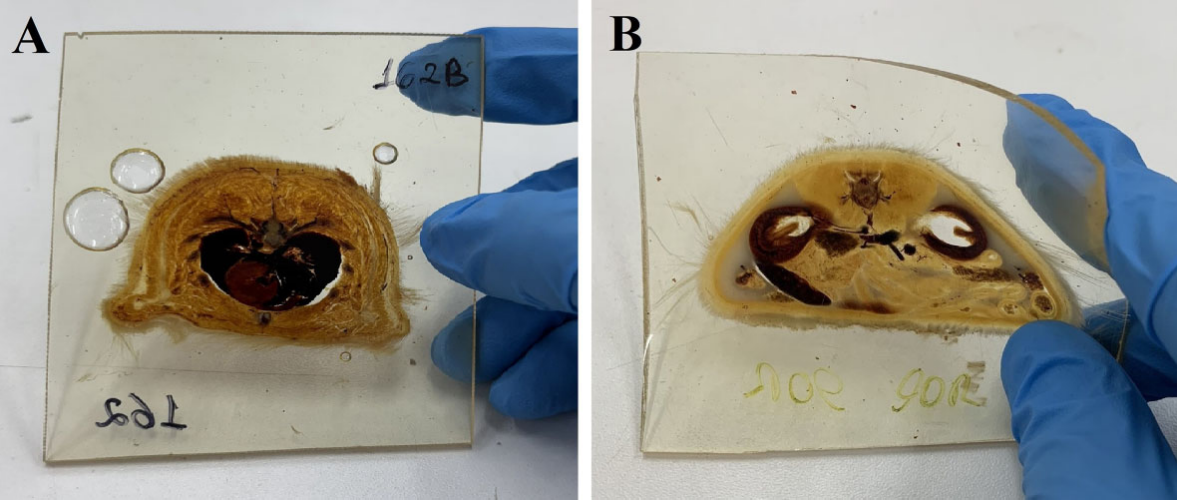
Wistar rat sections plastinated with epoxy E12 and hardener E1 (ratio 100:28) (**A**) and E48 with plasticizer and hardener E5 (ratio 65:35:28) (**B**) under slight tension, demonstrating the malleability of the E48 epoxy with the plasticizer.

In the first impregnation test with the E48 resin, the ratio of resin, plasticizer, and hardener of 65:35:28 m/m was used (sample six, Table 3). During curing, the impregnation mixture itself was used to assemble the sandwich, aiming to more closely replicate the Biodur^®^ protocol and maximize materials usage. This mixture provided a working time (before the gel state) of approximately 11 h and complete hardening of 60 h at room temperature (25±2°C). The disassembly of the cured slices was easy, without adhesion or spots with incomplete curing. However, after the addition of the plasticizer, the 2-mm slices showed a degree of malleability without fracturing (Figure 3), unlike the slices prepared with the reference epoxy, which were very rigid. Nonetheless, this characteristic may not be a problem, since it does not interfere with the visualization of the biological slices. From the first month of curing, the slices produced with the E48 resin used in the first test were noticeably yellowing under the mild lighting conditions of the Plastination Laboratory of UFES, along with slight arching. According to the literature, the use of plasticizers can accelerate the yellowing of the epoxy resin (12). In the same period, there was no sign of yellowing in the other experimental groups.

To produce more rigid plates as in the Biodur^®^ method, the same composition and proportion of the reactive mixture of the previous test (65:35:28 m/m for resin, plasticizer, and hardener) were used in the second and third tests for the forced impregnation stage. In the curing stage, a freshly prepared mixture of resin and 43% of hardener was used. The mixture was produced about 30 min before its use to increase its viscosity and avoid its extravasation from the assembled sandwich. The use of the plasticizer allowed satisfactory impregnation and complete curing of the samples. The plates formed with the alternative epoxy in tests two and three showed a greater number of small air bubbles than those of E12 and test one of the E48 resin. In addition, no arching of the section was noticed over time. The yellowing of the sections was noticeable only approximately 12 months after curing.

### Evaluation of tissue shrinkage

For the shrinkage analysis of the different types of biological tissues, 55 epoxy slices were evaluated, 27 were from the experimental group E12, and 28 from E48. 12 samples of each tissue and epoxy were measured for analysis. A total of 95 slices were disregarded based on the exclusion criteria for the analysis. More than one type of biological tissue may have been measured in the same section.

The Shapiro-Wilk and Levene tests showed that the data presented a normal distribution and homogeneity of variance for all groups and tissues (P>0.05). Therefore, Student's *t*-test and ANOVA were used.

Student's *t*-test was used to compare groups E12 and E48 for each biological tissue, and no statistically significant differences were found in the percentage of shrinkage between the groups for all tissues (P>0.05) (cardiac=[t(18)=1.455; P=0.163]; splenic=[t(18)=1.497; P=0.152]; hepatic: [t(18)=1.121; P=0.277]; nervous: [t(18)=1.371; P=0.187]; pulmonary: [t(18)=1.544; P=0.140]; renal: [t(18)=2.136; P<0.052]).

ANOVA was used to investigate differences between tissues within each group. No significant differences between the tissues were found in groups E12 (F=1.248; P=0.300) and E48 (F=0.761; P=0.582).

Table 4 shows the mean±SD percentage of shrinkage by area of the tissues, and these obtained from the percentage difference of the area (cm^2^) before impregnation and after curing, measured by the ImageJ software. Values were rounded to one decimal place. Shrinkage values in the two groups were close, with a difference of 1% between the highest and the lowest mean. Although no significant difference was found, the greatest tissue shrinkage was observed in hepatic tissue (10.0%±0.8) for E12 and in the hepatic and pulmonary tissue (9.2%±1.9 and 9.2%±1.1) for E48. The smallest shrinkage was found in cardiac tissue for both groups (9.0%±1 and 8.3%±1 for E12 and E48, respectively). In addition, E48 epoxy showed lower shrinkage compared to E12 for all analyzed tissues.

**Table 4:** Mean area before impregnation (Pre) and after curing (Post) and mean (SD) shrinkage percentage of the different tissues plastinated with E12 and E48 epoxy resins.

	Tissue	Pre (cm^2^)	Post (cm^2^)	Mean (%)	SD
E12	All	1.319	1.19	9.7	0.4
	Cardiac	1.248	1.131	9.0	1.0
	Pulmonary	0.633	0.569	9.9	1.0
	Renal	0.603	0.541	9.9	1.2
	Hepatic	3.793	3.422	10.0	0.8
	Splenic	0.586	0.529	9.7	1.0
	Nervous	1.050	0.950	9.5	1.3
E48	All	1.179	1.084	8.8	0.4
	Cardiac	0.795	0.729	8.3	1.0
	Pulmonary	0.905	0.820	9.4	1.1
	Renal	0.742	0.680	8.4	1.9
	Hepatic	3.237	2.947	9.0	1.9
	Splenic	0.362	0.330	8.8	1.3
	Nervous	1.036	0.945	8.8	0.9

The work of Monteiro et al. (11) demonstrated that different types of biological tissues tend to have different levels of shrinkage in the forced impregnation stage. The authors believe that the main factors for this difference are the biochemical composition of tissues (amount of water and lipids), the extracellular structure (type and amount of connective tissue of the extracellular matrix), and the tissue contact surface. However, this study did not find a statistical difference in shrinkage between the analyzed tissues, probably due to the large contact surface compared the tissue volume in the thin sections (2-3 mm), minimizing the effect of the other factors; as a result, the epoxy permeated the tissue very easily.

No study was found in the literature that measured tissue shrinkage in different types of tissues as presented in this study; only studies on the shrinkage of whole slices were found. However, there are a few studies on shrinkage in plastination with epoxy. Sora et al. (13) found that the average shrinkage of the E12 epoxy sections ranged from 4-6% in the forced impregnation/curing stage. The low shrinkage can be explained by the longer impregnation time (48 h) due to the addition of the Biodur^®^ AE10 compound, which extends curing time and decreases impregnation temperature (5°C). There is a well-established relationship about time or speed of impregnation and tissue shrinkage: the lower the speed of impregnation, the more efficient the acetone-to-polymer exchange in the tissues and, consequently, the lower the shrinkage (6,11,14,15).

As von Hagens stated, up to 10% of tissue shrinkage is inevitable in plastination. Shrinkage values that are lower than this threshold for the E12 technique are considered acceptable - and even desirable - serving as a proxy for a well-executed plastination process (16,17).

Although the viscosity of a polymer is known to be directly proportional to tissue shrinkage in the impregnation stage (11,15), the difference in viscosity of the tested epoxies (Table 2) was not sufficient to significantly affect shrinkage. As previously mentioned, this is likely due to the large contact surface and small thickness of the slices, which may have minimized tissue shrinkage.

In summary, E48 proved to be a plausible alternative to E12, with similar shrinkage rates between the two.

### Stereoscopic and confocal fluorescence microscopy

**Figure 4:**
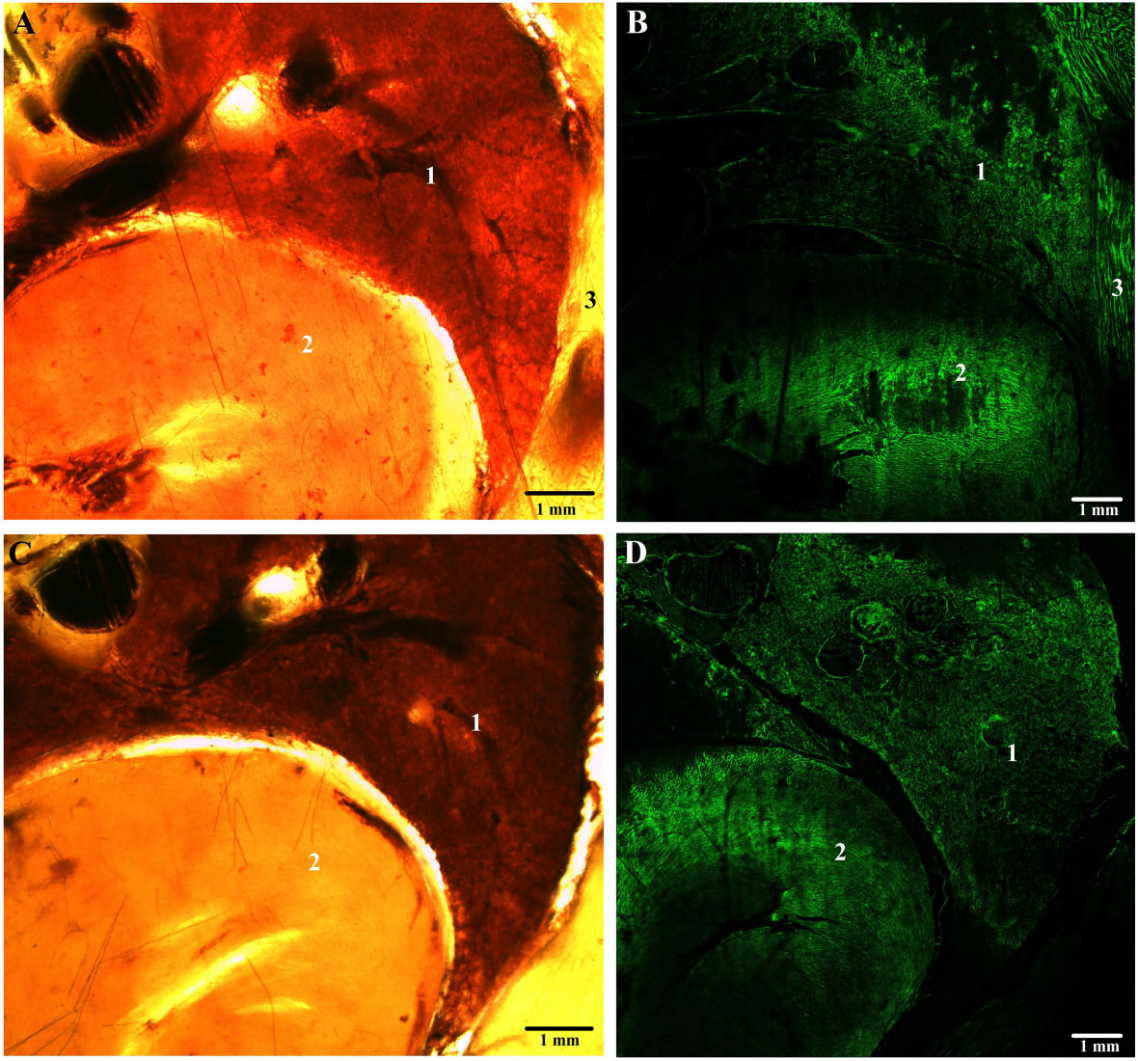
Cross-sections of Wistar rat chest plastinated with E12 epoxy (**A** and **B**) and E48 epoxy (**C** and **D**), seen by stereomicroscopy at 8× magnification (**A** and **C**) and confocal microscopy (**B** and **D**) at 10× magnification. 1=lung tissue; 2=heart tissue; 3=muscle tissue. Scale bar: 1 mm.

**Figure 5:**
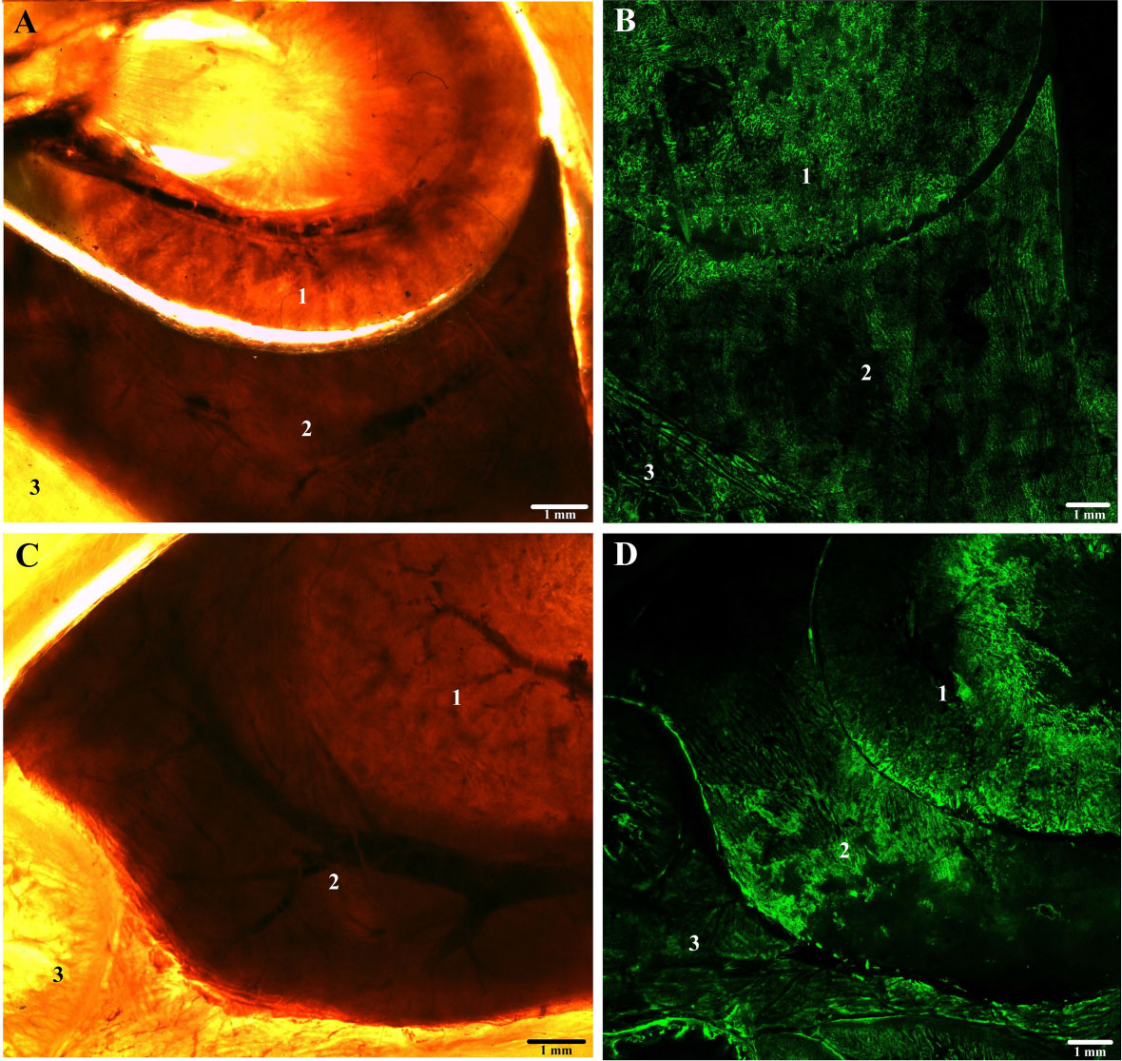
Cross-sections of Wistar rat abdomen plastinated with E12 epoxy (**A** and **B**) and E48 epoxy (**C** and **D**), containing intestinal tissue seen by stereomicroscopy (**A** and **C**) and confocal microscopy (**B** and **D**). 1=renal tissue; 2=liver tissue; 3=gut tissue. Scale bar: 1 mm.

**Figure 6:**
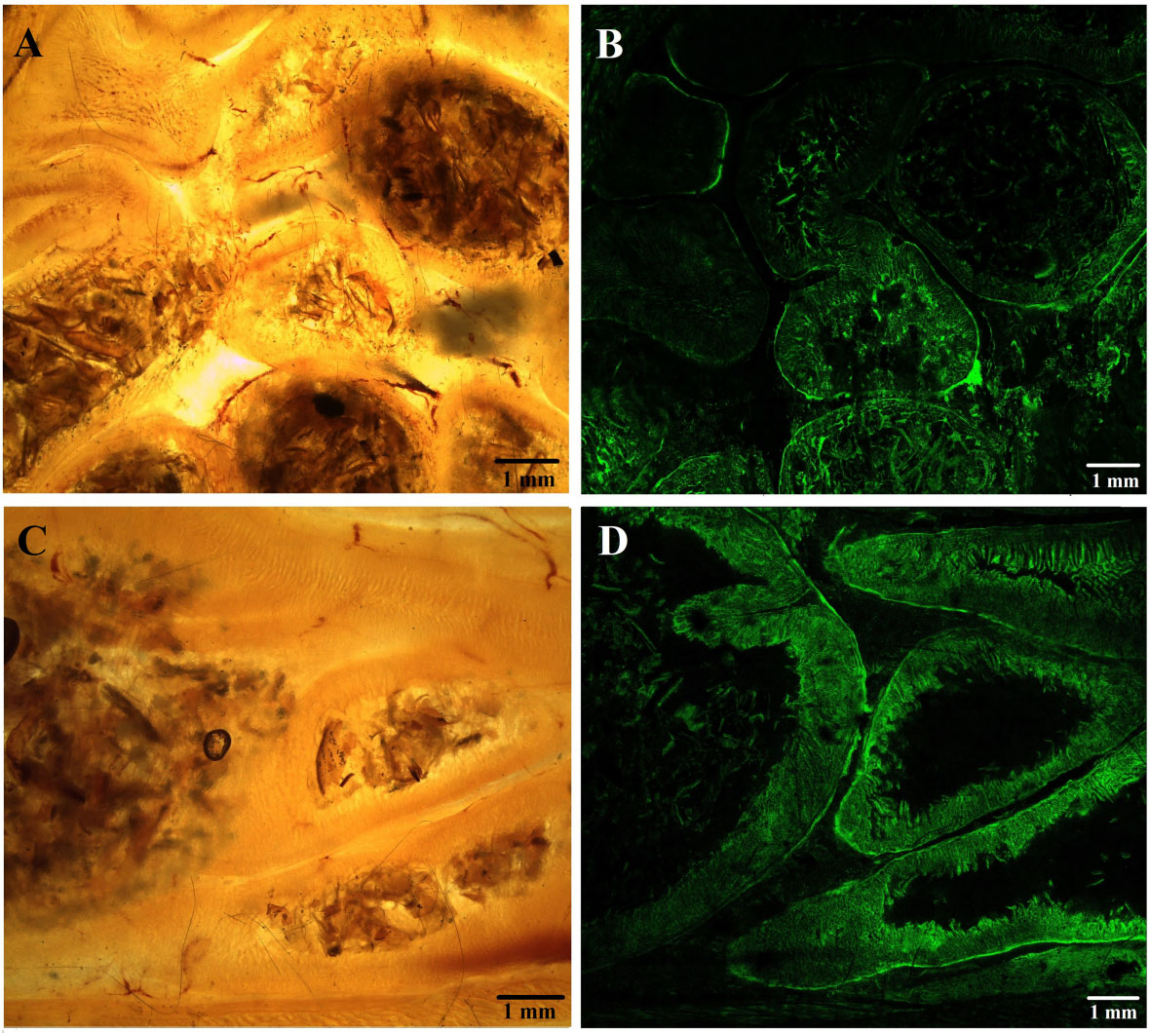
Cross-sections of Wistar rat abdomen plastinated with E12 epoxy (**A** and **B**) and E48 epoxy (**C** and **D**) seen by stereomicroscopy (**A** and **C**) and confocal microscopy (**B** and **D**). Scale bar: 1 mm.

Figures 4, 5, and 6 show the comparison between plastinated tissues with E12 and E48 in stereoscopic and confocal microscopy with autofluorescence at 488 nm wavelength.

Microscopy images revealed high-quality visualization of tissue organization. Tissues limits and macroscopic structures were clearly identified, and cavitary structures such as blood vessels and bronchioles were evident and easily observed (Figures 4 and 5).

The plastination process results in endogenously autofluorescent connective tissue observable at 488 nm excitation wavelength, especially collagen and elastin (18). From our qualitative and subjective analysis, a higher autofluorescence and contrast was observed in the tissues plastinated with E48 epoxy. In these samples, the histological structures and the boundaries between the tissues were better defined. This could be explained by a higher refractive index of E48, which has a lower interference in the capture of autofluorescence images. It is known that one of the preponderant factors for the semi-transparency of slices plastinated with epoxy is the higher refractive index of the resin (1,551, on average) compared to that of water (1,333) (19).

In general, no distortions or other significant morphological changes were observed by fluorescence microscopy in the epoxy-plastinated tissues, with the typical structure of each tissue preserved. The tissues were very similar between experimental groups.

**Figure 7:**
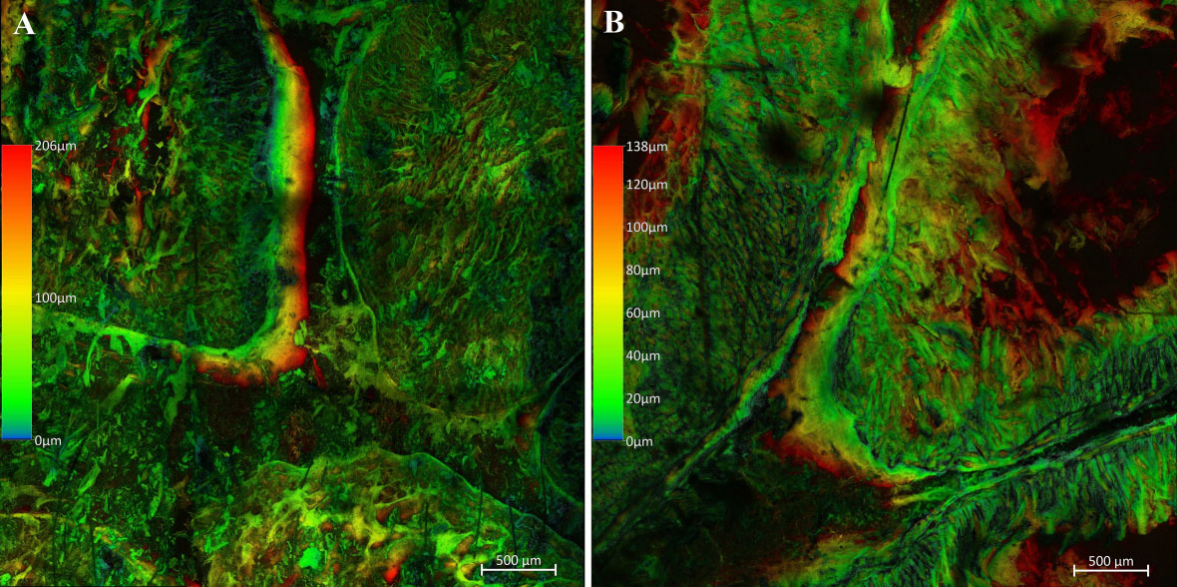
Z-stack images of Wistar rat gut plastinated with E12 (**A**) and E48 (**B**) epoxy showing the depth of the analyzed regions in micrometers (μm). Scale bar: 500 μm.

Figure 7 shows Z-stack images of the intestinal tissue plastinated with E12 and E48 epoxies obtained with the confocal microscope. The Z-stack images show in high quality and detail the tissue architecture of the intestine formed by connective tissue at different levels of the plastinated sections, and the groups cannot be visually distinguished. Using this tool, it was also possible to perform 3D reconstruction of the area captured by the microscope.

Microscopy is an important tool in the investigation of biological tissues from plastinated epoxy sections (18,20,21), especially for anatomical descriptions and 3D tissue reconstruction. The accuracy of 3D reconstructions from material plastinated with epoxy is comparable to those obtained from computed tomography or magnetic resonance imaging, making it a valuable option for morphometric analyses (20,22,23). The literature includes several investigative studies in which confocal and stereoscopic microscopy were used for basic anatomical descriptions and applied clinical research, particularly in structures consisting mainly of connective tissue, such as ligaments and fascia. The work of Liugan et al. (24), aimed to reveal the configuration of fibrous structures within the optic canal and their relationship to the optic nerve, intracranial subarachnoid space (SAS), and ophthalmic artery to test the hypothesis that cerebrospinal fluid does not flow continuously between the intracranial SAS and the SAS within the optic nerve sheath. The work of Nash et al. (21) aimed to investigate the three-dimensional organization of the connective tissues in the anterior triangle of the neck to assess whether the lining layer of the deep cervical fascia exists. In both studies, the main investigative tool was confocal microscopy of epoxy-plastinated specimens. Therefore, our findings indicate that applied clinical research can be conducted with E48.

Given the results found, the use of E48 epoxy proved to be effective in the plastination of biological tissues and an alternative to the reference epoxy (E12) in macro- and microscopic evaluations. Furthermore, from an economic point of view, the E48 impregnation mixture costs 50% less than E12 (EUR/BRL quoted at 6.35).

## Conclusion

The alternative epoxy E48 had excellent performance in plastination, shrinkage analysis, and microscopy of Wistar rat sections compared to the Biodur^®^ E12 control group. Visually, the plastinated sections (2-3 mm) of the two groups did not show differences in terms of color, crystallinity, and appearance. In the analysis of tissue shrinkage by area, the tested epoxies did not show a significant difference, with shrinkage of 8.8 to 9.7%. In addition, the cost of E48 epoxy in 2024 was less than half that of E12. Chemically, both epoxies are similar and based on diglycidyl ether of bisphenol A. Finally, plastination with epoxy in flexible plates containing a plasticizer resulted in new products and possibilities not yet available in the global market.
